# ScRNA-seq and ST-seq in liver research

**DOI:** 10.1186/s13619-022-00152-5

**Published:** 2023-02-03

**Authors:** Jia He, Chengxiang Deng, Leonard Krall, Zhao Shan

**Affiliations:** State Key Laboratory of Conservation and Utilization of Bio-resources in Yunnan and Center for Life Sciences, School of Life Sciences, Yunnan University, Kunming, 650091 China

**Keywords:** Liver development, Liver regeneration, Liver disease, Single-cell RNA sequencing, Spatial transcriptome sequencing

## Abstract

Spatial transcriptomics, which combine gene expression data with spatial information, has quickly expanded in recent years. With application of this method in liver research, our knowledge about liver development, regeneration, and diseases have been greatly improved. While this field is moving forward, a variety of problems still need to be addressed, including sensitivity, limited capacity to obtain exact single-cell information, data processing methods, as well as others. Methods like single-cell RNA sequencing (scRNA-seq) are usually used together with spatial transcriptome sequencing (ST-seq) to clarify cell-specific gene expression. In this review, we explore how advances of scRNA-seq and ST-seq, especially ST-seq, will pave the way to new opportunities to investigate fundamental questions in liver research. Finally, we will discuss the strengths, limitations, and future perspectives of ST-seq in liver research.

## Background

The liver is a multifunctional organ simultaneously acting as a store house, a manufacturing hub, and a processing plant. Excess nutrients that are not immediately required by the body are stored in the liver in the form of glycogen, which is released back into the blood stream during fasting (Vdoviakova et al. 2016; Han et al. 2016). The liver also works as a manufacturing hub, producing a wide array of circulating substances, such as coagulation factors, hormones, vitamin D, and most importantly bile (Boyer and Soroka 2021). Last but not least, the liver is a processing plant, as the majority of toxic substances such as alcohol, drugs, and pesticides are metabolized into non-toxic substances in the liver (Hildebrandt et al. 2021). With all these diverse roles, the question arises as how can the liver perform so many different processes simultaneously?

**Fig. 1:**
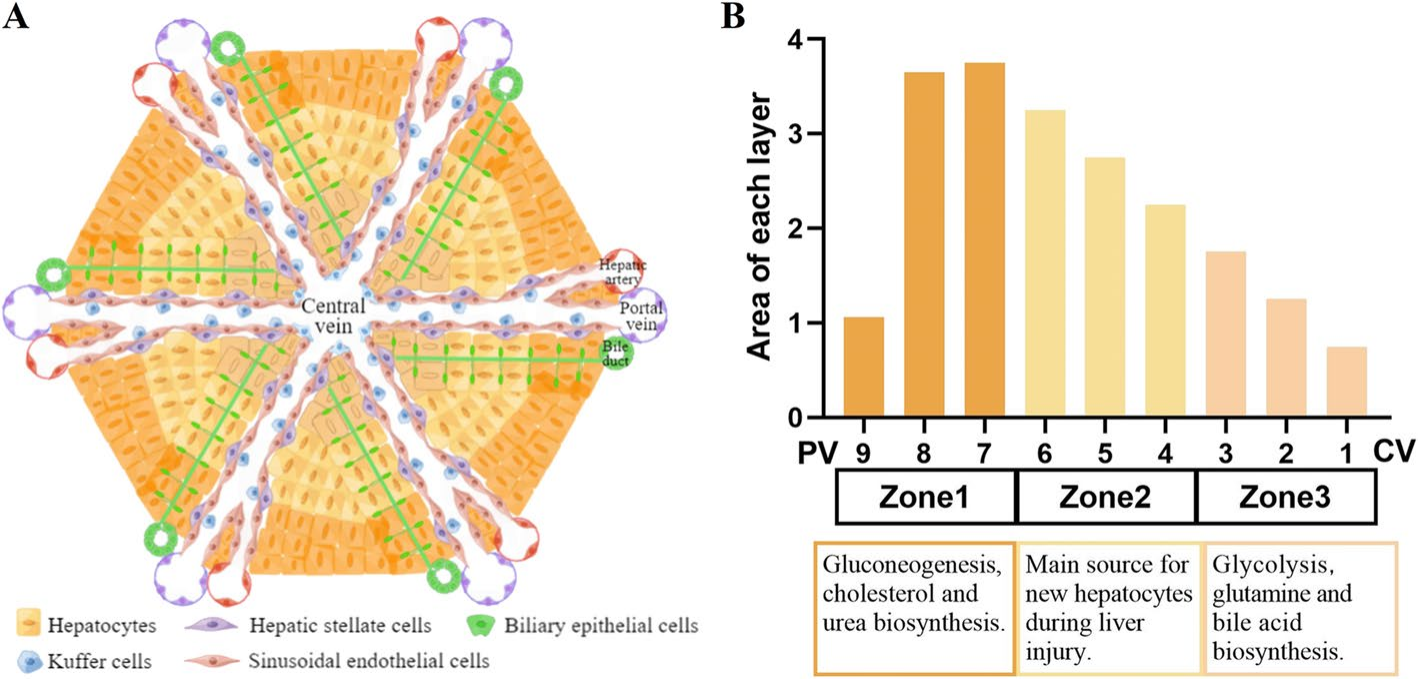
Liver structure and zonation. **A** Schematic diagram of hepatic lobule and zonation. **B** A summary of hepatic zonation and its main function. Adapted from Halpern K.B.et al. 2017

One reason the liver can handle so many disparate processes is its complex architecture. Structurally, the liver contains numerous repeating hexagon units, termed liver lobules (Itzkovitz 2019). Lobules are composed of a portal triad, composed of a portal vein, bile duct, and a hepatic artery, with hepatocytes in linear rows surrounding a capillary network. In the center of each liver lobule is the central vein. The hepatic portal vein connects the digestive tract with the liver and transports various nutrients and toxins from the gut to the liver, while the hepatic artery carries oxygenated blood from the heart to the liver. When the hepatic artery and portal vein converge in the liver, blood with nutrients and oxygen is collected into capillaries called hepatic sinuses in each lobule. The blood flows through each lobule towards the central vein and all the central veins finally merge into the inferior vena cava to return the blood to the heart. The bile duct is responsible for collecting bile synthesized by hepatocytes. In contrast to the flow of blood, bile flows backward from the center of the lobule and drains in the peripheral portal bile duct. Along the axis from the portal area towards the central vein, nutrients and oxygen from the blood are continuously absorbed by cells on both sides, thus forming a gradient axis of oxygen and nutrient exposure. This gradient axis dictates that metabolic processes with different oxygen requirements are asymmetrically distributed. Based on this fact, Rappaport first delineated the zones of the liver into the periportal zone, the midzone, and the pericentral zone (Rappaport 1958; Rappaport 1973; Rappaport 1976). Each liver lobule was further divided into 9 layers by reconstruction of spatial division of labor using scRNA-seq (Halpern et al. 2017). Along the axis from the central vein to the portal vein, each liver lobule is divided evenly into nine concentric rings: layers 1–3 are close to central vein, layers 4–6 are mid-layers, and layers 7–9 are around the portal area. Cells residing in different radial layers sub-specialize different metabolic functions according to their distance from the central vein or portal vein. This phenomenon is called “liver zonation” (Halpern et al. 2017; Katz 1982). Hepatocytes residing in each zone are transcriptionally different, especially in the expression of metabolism-related genes (Gebhardt and Matz-Soja 2014). For example, glutamine synthetase is specifically expressed within two layers of hepatocytes surrounding the central vein, whereas ureagenesis is exclusively confined to periportal areas (Jungermann 1988). Periportal zone (also called zone 1) is considered to play a central role in gluconeogenesis, cholesterol and urea biosynthesis, while the pericentral zone (also called zone 3) is considered to play an important role in glycolysis, glutamine and bile acid biosynthesis (Paris and Henderson 2022). The midzone (also called zone 2) is the main source of new hepatocytes during homeostasis and regeneration *in vivo (*Wei et al*. *2021*)*. Overall, the complex architecture of the liver, especially liver zonation, shows how the liver can play multiple roles simultaneously (Fig. 1).

The other reason the liver can handle so many disparate processes might be the diverse cell types in liver, which interact with each other and play distinct roles during liver homeostasis and disease. The liver is composed of parenchymal cells and non-parenchymal cells (NPCs). Parenchymal cells are mainly hepatocytes, and account for 90% of liver mass (Gissen and Arias 2015). NPCs include cholangiocytes, sinusoidal endothelial cells (LSECs), hepatic stellate cells (HSCs), Kupffer cells (KCs), as well as others (Aizarani et al. 2019). Cholangiocytes are biliary epithelial cells, which are mainly responsible for regulating bile flow in the liver (Jones et al. 2015). LSECs account for about 50% of NPCs and constitute the blood vessel walls of the liver microcirculation system (Gracia-Sancho et al. 2021). KCs are liver resident macrophages and maintain liver homeostasis by functioning as phagocytes (Amirali Kiyani 2015). Studies have shown that macrophages are heterogeneous and function differently under different microenvironment (Mantovani et al. 2004; Gordon and Taylor 2005; Leroux et al. 2012). HSCs are the main source of extracellular matrix and are target cells of various fibrosis factors (Higashi et al. 2017; De Smet et al. 2021). Normally, HSCs are quiescent, but they become activated when liver injury occurs (De Smet et al. 2021). Along with the cell types described above, immune cells from blood circulation also contribute to liver homeostasis and disease such as monocytes, eosinophils, infiltrating macrophages, neutrophils, and natural killer T cells. More interestingly, an increasing number of various immune cells are recruited into the liver during liver disease, either exacerbating or alleviating disease progression (Kolodziejczyk et al. 2020; Chauhan et al. 2020; Shan et al. 2021). In general, the vast array of cells in the liver follow the principle of “parenchymal cells perform, non-parenchymal cells trigger regulation,” and they all jointly regulate liver homeostasis and disease (Ding et al. 2016).

Although the heterogenicity of liver cells was discovered long ago, studies on cell fate or crosstalk have been severely hindered by technical limitations. Single cell transcriptomics have largely expanded our understanding of cell heterogeneity. Recent ST-seq advances further allows for the characterization of cell heterogeneity in situ. Studies on the liver generally follow one of three directions: liver development, liver regeneration, and liver disease. In this review, we first give a general introduction of scRNA-seq and ST-seq. Secondly, we explore how advances of scRNA-seq and ST-seq, especially ST-seq, open new opportunities to investigate fundamental questions in liver development, regeneration, and disease, respectively. Finally, we will discuss the strengths, limitations, and future perspectives of ST-seq in liver research.

## A general introduction to scRNA-seq and ST-seq

ScRNA-seq is used to examine RNA expression from an individual cell. Therefore, scRNA-seq enables one to understand the cellular transcriptome, and therefore possibly the function of each cell under specific circumstances. To get scRNA-seq results, we must go through sample preparation, sequencing, and data analysis. Sample preparation includes three steps: cell isolation, single cell preparation, and library construction. Sequencing platforms include Ilumina 10 x Genomics chromium (Porreca et al. 2007), Roche pyrosequencing (Dong et al. 2011), BD Rhapsody (Gao et al. 2020), as well as others. Data analysis is highly dependent on software. Commonly-used analysis software include Seurat (Satija et al. 2015), monocle (Qiu et al. 2017), Scanpy (Wolf et al. 2018), galaxy (Goecks et al. 2010), etc. High throughput scRNA-seq data further enables advanced analysis, such as inference of cellular function or cell trajectory analysis, as well as others. Inference of cellular function can be done using enrichment analysis, Gene Ontology (GO) analysis, or Kyoto Encyclopedia of Genes and Genomes (KEGG) pathway analysis, to mention but a few. These analyses use differentially expressed genes to predict potential associated signaling pathways. Cell trajectory analysis can be used to predict cell differentiation or development progression by comparing gene expression levels among various cells. Pseudo time analysis and RNA velocity are two main ways to perform cell trajectory analysis. As cells differentiate, they undergo a process of transcriptional reorganization, with changes in gene expression. Pseudotime analysis (Hou et al. 2021) infers the course of cell differentiation over time by building a pseudotime trajectory of changes in gene expression among different cell populations. RNA velocity (Bergen et al. 2020) speculates the dynamic differentiation of cells by comparing the ratio of spliced and unspliced mRNA. Newly proliferated cells possess a higher ratio of unspliced mRNA than spliced mRNA.

ScRNA-seq makes it possible to analyze RNA expression at the single-cell level (Ziegenhain et al. 2017). However, scRNA-seq requires cell isolation from tissues, and during this process they might lose their original spatial identity. ST-seq overcomes this weakness by capturing RNA information and performing sequencing in situ (Marx 2021). Sample preparation for ST-seq is based on spatial barcoding technology and includes three major steps (Williams et al. 2022). First, frozen tissue is sectioned and immobilized on a chip with preset barcodes. Secondly, the tissue is permeabilized to release RNA, which is captured by poly-dT oligos preset on the chip array. Third, once bound to the chip, RNA is reverse transcribed and amplified by PCR for sequencing. Among these steps, permeabilization is the key step. It requires optimization to preserve maximum RNA and cell morphology. Although ST-seq can preserve spatial information *in situ*, the most widely used ST-seq technology still cannot reach single-cell resolution. Therefore, some researchers prefer to combine scRNA-seq with ST-seq to examine cellular heterogeneity.

## ScRNA-seq and ST-seq in the study of liver development

The liver is a multifunctional organ with various cells derived from multiple lineages. Therefore, where and how the early liver emerges during organogenesis is one of the big questions in the field of liver development. The liver parenchyma is composed of hepatocytes and bile duct epithelial cells (BECs). However, the cellular origin of liver parenchyma during embryonic development has been controversial. A type of hepatobiliary hybrid progenitor (HHyP) was discovered in human fetal liver by scRNA-seq technology (Segal et al. 2019). The HHyP is characterized by epithelial cellular adhesion molecule (EpCAM)^+^ located at the ductal plate in the fetal liver, and it possess a transcriptional profile distinct from hepatocytes and BECs. Furthermore, they confirmed that fetal HHyP possesses a hybrid gene expression signature of both hepatic and biliary lineage. In addition to liver parenchyma, scRNA-seq and ST-seq have been applied to study the origin of NPCs. Fifteen scRNA-seq libraries containing 45,334 single-cell transcriptomes from embryonic day (E)7.5, were integrated to trace cell lineage (Lotto et al. 2020). Harmony for batch correction and Palantir for pseudotime analysis were employed to predict cell plasticity and bifurcation points during early liver development. Their data showed that LSECs diverge from vascular endothelial cells by E8.75 with a distinct transcriptional profile. As well, they presented distinct spatial temporal distributions for two distinct mesothelial cell types and early HSCs. Hepatoblast specification and migration were also revealed using these transcriptional profiles. Moreover, they indicated that some cell crosstalk occurs during the early formation of the sinusoid. Overall, these results generated a comprehensive map of a primitive liver cell lineage from the endoderm and mesoderm.

The liver is structurally and metabolically zonated, which forms the basis for normal liver function. Researchers have been curious to know what signaling pathway regulates zonation and how genes are transcriptionally active in specific zones. With the introduction of scRNA-seq and ST-seq into liver research, our understanding about liver zonation has been further expanded. The whole transcriptome of murine liver by scRNA-seq was measured and their lobule equivalents based on landmark genes were inferred (Halpern et al. 2017). About half of liver genes are zonated along the gradient axis, such as Hepcidin Antimicrobial Peptide (Hamp), Hepcidin Antimicrobial Peptide (Hamp2), Insulin Like Growth Factor Binding Protein 2 (Igfbp2), major urinary protein 3 (Mup3), and cytochrome P450 family 8 subfamily B member 1 (Cyp8b1). Wnt signaling is essential for the formation of pericentral zonation while Ras signaling is required for the formation of periportal zonation. Genes related to oxidative phosphorylation are highly expressed around the periportal area, while genes related to detoxification pathways, bile acid biosynthesis and proteasome composition are highly expressed around the central vein. To add global transcriptomics with spatial information across lobular units, spatial heterogeneity in the liver was further explored using ST-seq (Hildebrandt et al. 2021). ST-seq data from 8 wild-type mice were integrated by canonical correlation analysis (CCA) and 6 clusters were obtained by unsupervised clustering. Following examination of each cluster by differential gene expression analysis (DGEA), scRNA-seq data were mapped back to ST-seq tissues using stereoscopy imaging. Spatial isolation was found to be existed between hepatocytes around the portal vein and the central vein through correlation analysis. Moreover, the gene transcriptomes from these two subsets of hepatocytes are negatively correlated with their spatial distribution. Module score analysis of each cluster by Seurat showed that gene expression of each cluster changes in a gradient manner along the axis of the hepatic lobule. Together, these data confirmed that spatial heterogeneity in each liver lobule is shaped by differential transcriptomes along the hepatic lobule axis. Along the metabolic gradient axis, not only hepatocytes but also LSECs are zonated. LSECs also display spatial and molecular heterogeneity along the axis of the liver lobule. The proteome landscape of the liver endothelium was generated by combining spatial cell sorting, scRNA-seq, and quantitative proteomics/phosphoproteomics (Inverso et al. 2021). Tyrosine phosphorylation was found to be a unique zonated process and that Tie1 phosphorylation acts specifically around the central vein area as a signature of the LSECs located there. Overall, the studies above jointly corroborated that liver zonation is regulated by Wnt signaling and that many genes are zonated along the gradient axis.

Scientists have always been interested in the cellular composition of the liver. A human liver cell atlas was constructed by performing scRNA-seq on 10,000 cells from normal human liver tissue (Aizarani et al. 2019). Various previously unknown subpopulations of LSECs, KCs, and hepatocytes were identified. Moreover, they showed that the EPCAM^+^ population is heterogenous, and is expressed in hepatocytes, cholangiocytes, and a TROP2^int^ progenitor population. The HSCs have been thought as a homogenous cell population playing an important role in liver fibrosis. Two subpopulations of HSCs were identified by supervised clustering after independent components analysis dimensionality reduction: portal vein-associated HSCs (PaHSCs, Adamtsl2^hi^HSC) and central vein-associated HSCs (CaHSCs, NGFR^hi^ HSC) (Dobie et al. 2019). More importantly, they found that CaHSCs are the main collagen-producing cells in a carbon tetrachloride (CCl4) -induced liver fibrosis mouse model. Blocking lysophosphatidic acid receptor 1 (Lpar1), which is specifically expressed in CaHSCs, can inhibit liver fibrosis in nonalcoholic steatohepatitis mouse model. Overall, they identified collogen-producing HSCs and provided a precise therapeutic target for liver fibrosis. Although scRNA-seq has greatly expanded our understanding of cell heterogeneity in the liver, controversies have also been raised due to the complex analysis process. Two populations of KCs were claimed to be identified via scRNA-seq technology (Blériot et al. 2021). To better understand the two subsets of KCs, they performed another scRNA-seq method (SMARTseq2) that has a higher resolution on pre-sorted KCs. The results confirmed that two subpopulations of KCs exist in murine liver: a major CD206^lo^ESAM^−^ population (KC1) and a minor CD206^hi^ESAM^+^ population (KC2). Interestingly, KC2 highly expresses mannose receptor C-type 1 (Mrc1), platelet and endothelial cell adhesion molecule 1 (Pecam1), kinase insert domain receptor (Kdr), and lymphatic vessel endothelial hyaluronan receptor 1 (Lyve1), which are also highly expressed in LSECs. However, soon after this study was published, another study suggested that there is only one population of KCs and that the second population of KCs in Bleriot’s study might be LSECs/KCs doublets by single-cell CITE-seq and 3D reconstruction (Guilliams et al. 2022). Nonetheless, while sequencing may provide us with enormous amounts of data, more caution must be taken with the data analysis and conclusions (Table 1).

## ScRNA-seq and ST-seq in the study of liver regeneration

Although the remarkable regenerative capacity of the liver has been well established, the cell types or liver zonation that contribute to liver regeneration is still an active area of research (Paris and Henderson 2022). Whether hepatocyte progenitor cells (HPCs) exist or how much they contribute to liver regeneration are still being debated. Pericentral Axin2^+^ (Wang et al. 2015) or periportal Sox9^+^ (Font-Burgada et al. 2015) or Mfsd2a^+^ (Pu et al. 2016) hepatocytes have been proposed to be HPCs, serving as sources of new hepatocytes following liver injury. However, several independent groups have used varied approaches and have shown that hepatocytes, irrespective of zonal position, possess regenerative capacity (Matsumoto et al. 2020; Sun et al. 2020; Chen et al. 2020; Ang et al. 2019). Most recently, two studies suggested that liver homeostasis is preferentially maintained by Zone 2 hepatocytes (Wei et al. 2021; He et al. 2021). ScRNA-seq and ST-seq have also been applied to clarify this issue. The differential outcomes of Wnt signaling-mediated proliferation between Axin2+ hepatocytes and Axin2^+^ intestinal stem cells have been addressed by ST-seq (Sun et al. 2021). Although chromatin accessibility is the same in both cells, ZNRF3 and RNF43 fine-tune the activity of WNT-β-Catenin in the liver, thereby preventing hepatocyte proliferation but maintaining metabolic zonation. These results added another layer of evidence that pericentral Axin2^+^ hepatocytes may not serve as HPCs. Recently, scRNA-seq, bulk RNAseq, ST-seq, and smFISH imaging have been integrated to study liver regeneration following acetaminophen (APAP)-induced pericentral liver injury (Ben-Moshe et al. 2022). The data indicated that hepatocytes throughout the lobule proliferate almost equally to generate missing cells. Mitotic pressure promotes hepatocytes in Zone 2 to replace the necrotic central area. All hepatocytes go through a transcriptional and metabolic reprograming, which contributes to reconstruction of liver zonation. Along this process, NPCs serve as zone-specific cues to instruct regeneration. Although advanced sequencing methods have been used to study what cells contribute to liver regeneration, the existence of progenitor cells is still controversial. As mentioned above, a TROP2^int^ progenitor population was identified by performing scRNA-seq on 10,000 cells from normal human liver tissue (Aizarani et al. 2019). Furthermore, the study also showed that TROP2^int^ cells have the potential to form liver organoids in vitro. Clearly more research is necessary to conclusively determine whether HPCs exist. Perhaps the potency of HPCs to generate new hepatocytes depends on location and microenvironmental cues.

Microenvironmental cues have been suggested to play an important role in liver regeneration. However, due to technical limitations, this research field remains elusive. Apart from zonated, LSECs also contribute to hepatocyte regeneration via a Tie2/Wnt signaling pathway by scRNA-seq technology (Inverso et al. 2021). It has been revealed that Wnt production is dependent on Tie1 in LSECs and Wnt is critical to sustain the liver regenerative niche and to restore liver mass after injury (Table 1).

## ScRNA-seq and ST-seq in the study of liver disease

The complex cellular composition of the liver makes it hard to decipher the molecular mechanisms underlying the progression of liver disease. An increasing number of studies have adopted scRNA-seq and ST-seq to decode the changes in cellular composition during liver disease. A cellular atlas for acute liver injury has been generated using APAP and thioacetamide (TAA) -induced liver injury mouse models (Kolodziejczyk et al. 2020). 56,527 single-cell transcriptomes were characterized and multiple previously uncharacterized cell subsets for HSCs, LSECs, KCs, monocytes, and neutrophils were identified. Interestingly, three cell types were uncovered to be activated during acute liver failure (ALF): ALF-activated stellate cells (AAs), ALF-activated endothelial cells (AAe), and ALF-activated Kupffer cells (AAk). GO analysis further indicated that AAs and AAk might be associated with cell cycle arrest, while AAe might be associated with vascular remodeling. Moreover, MYC transcription is highly active in these cells. Inhibition of MYC via inhibitors prevented activation of these cells and reduced ALF. Taken together, these results indicate that scRNA-seq may provide detailed cellular decoding and possibly enable the finding of pathway-specific therapeutic targets possible. Single-cell transcriptome between normal livers and liver cancers have been compared to identify molecular changes at single-cell level during the development of liver cancer (Wang et al. 2021). By analyzing the transcriptome changes of each cell population, two populations of fibroblasts were uncovered. One subset expressed Sparc like 1 (Sparcl1) and the other subset expressed gap junction protein alpha 4 (Gja4). Moreover, the former fibroblast subset is associated with reduced vascular invasion and increased patient survival. Their study provided new insights into cell atlas alteration, especially for the Sparcl1^+^ population of fibroblasts in liver cancers.

Although various cell types have been identified in the liver, how these cells are organized in their distinct microenvironments remain poorly understood. Multi-omic datasets by single-cell CITE-seq, single-nuclei sequencing, spatial transcriptomics, and spatial proteomics were integrated and a spatial proteogenomic atlas of the healthy and obese human and murine liver was drawn (Guilliams et al. 2022). All liver cells were discriminated and localized, including a population of lipid-associated macrophages (LAMs) at the bile ducts. After aligning the atlas across seven species, KCs and LAMs are found to be conserved. In addition, LAMs are induced by local lipid exposure, which leads to their induction in the steatotic liver. Moreover, the development of KCs depends on their interaction with HSCs through the evolutionarily conserved ALK1-BMP9/10 axis. Overall, the data demonstrated that the phenotype of hepatic macrophages are driven by evolutionary conserved and microenvironmental signals. ST-seq has been performed on liver tissues from seven patients with primary liver cancers (PLCs), including five with hepatocellular carcinomas (hcc-1, 2, 3, 4, 5), one with hepatocellular and cholangiocarcinoma (chc-1), and one with intrahepatic cholangiocarcinoma (icc-1) (Wu et al. 2021). Liver tissues were divided into three regions: a normal region, the tumor region, and a stromal region. The enrichment score for each region showed that the stromal region is full of fibroblasts while the tumor region is infiltrated with immune cells. After integrating ST-seq data with scRNA-seq data for multimodal intersectional analysis (MIA), different PLCs are spatially heterogenous. Hcc-1,3,4 have high spatial continuity and intact capsule with more stromal and immune cells, which might indicate that the tumor capsule prevents immune cell infiltration and thus redistributes the immune cells around the cancer. As well, the tumor capsule affects intratumor spatial cluster continuity and transcriptome diversity. Region-specific changes of LSECs in liver cirrhosis were revealed by scRNA-seq technology (Su et al. 2021). Based on previously reported LSECs landmark genes, three clusters of LSECs were mapped into zone 1, 2, and 3 respectively. In comparison with the control group, genes associated with capillarization of LSECs and the extracellular matrix are mostly upregulated in LSECs of Zone 3. In addition, in cirrhotic mice, LSECs of Zone 3 also showed a decreased expression of endocytic receptor. Nitric oxide production-related transcription factors such as Kruppel-like factor − 2 (Klf2), Kruppel-like factor − 4 (Klf4), and Activation protein 1 (AP-1) are down-regulated in LSECs in all zones in cirrhotic mice, indicating increased intrahepatic vascular resistance. Together, application of ScRNA-seq and ST-seq have deepened our understanding about the effects of microenvironmental cues on liver cells during liver disease (Table 1).

**Table 1:** A summary of scRNA-seq and ST-seq applied in liver research

Classification	Methods	Major Findings	Reference
Liver development	scRNA-seq	Hepatobiliary hybrid progenitor (HHyP) cells at the ductal plate were found in human fetal liver, which own a hybrid transcriptome of both hepatic and biliary lineage.	Segal et al. 2019
	scRNA-seq ST-seq	A comprehensive map of primitive liver cell lineage was generated from the endoderm and mesoderm.	Lotto et al. 2020
	scRNA-seq	The whole transcriptome of murine liver was sequenced and analyzed.	Halpern et al. 2017)
	ST-seq	Spatial heterogeneity in each liver lobule shaped by differential transcriptomes along gradient was confirmed.	Hildebrandt et al. 2021
	Spatial cell sorting scRNA-seq proteomics /phosphoproteomics	Tyrosine phosphorylation is a unique zonated process and Tie1 phosphorylation acts specifically around central vein area as a signature of LSECs located around central vein.	Inverso et al. 2021
	scRNA-seq	Various previously unknown subpopulations of LSECs, KCs and hepatocytes were found. Moreover, a TROP2^int^ progenitor population was suggested.	Aizarani et al. 2019
	scRNA-seq	Two subpopulations of HSCs were found. One population mainly produce collagen and are associated with liver fibrosis.	Dobie et al. 2019
	scRNA-seq	Two subpopulations of KCs (a major CD206^lo^ESAM-population (KC1) and a minor CD206^hi^ESAM^+^ population (KC2)) were suggested.	Blériot et al. 2021
	scCITE-seq 3D reconstruction	This study suggested that there is only one population of KCs and the second population of KCs in previous study might be LSECs/KCs doublets.	Guilliams et al. 2022
	ST-seq ATAC-seq	ZNRF3 and RNF43 fine-tune activity of Wnt signaling-mediated proliferation in Axin2^+^ hepatocytes, thereby prevent hepatocytes proliferation but maintain metabolic zonation. This study suggested that Axin2+ hepatocytes are not likely hepatocyte progenitor cells.	Sun et al. 2021
Liver regeneration	scRNA-seq bulk RNA-seq ST-seq	Hepatocytes throughout the lobule proliferate almost equally to generate missing cells under mitotic pressure. Along this process, NPCs serve as zone-specific cues to instruct regeneration.	Ben-Moshe et al. 2022
	scRNA-seq	A TROP2^int^ progenitor population from normal human liver tissue was identified, which have a potential to form liver organoid in vitro.	Aizarani et al. 2019
	scRNA-seq	LSECs contribute to hepatocytes regeneration via a Tie2/Wnt signaling pathway.	Inverso et al. 2021)
	Seq-Scope	Spatial single-cell analysis of hepatocytes confirmed hepatocyte zonation and NPCs patial distribution was identified. Antioxidant genes in murine liver with early onset liver failure display zonated periportal-expression profile. With dead hepatocytes as the center, inflammatory macrophages and hep_injured cells are arranged around the periphery in turn.	Cho et al. 2021
	scRNA-seq	Mice with acute liver injury present with new subpopulations of HSCs, LSECs, KCs, monocytes, neutrophils, and acute liver injury can be alleviated by inhibition of MYC.	Kolodziejczyk et al. 2020
Liver disease	ST-seq	Two populations of fibroblasts (Sparcl1^+^ population and Gja4^+^ population) were indicated. Sparcl1^+^ population is associated with reduced vascular invasion and increased patient survival.	Wang et al., 2021
	scCITE-seq sn-seq ST-seq	A spatial proteogenomic atlas of the healthy and obese human and murine liver was generated. A population of lipid-associated macrophages (LAMs) at the bile ducts were identified, which was induced by local lipid exposure in steatotic liver. The development of KCs is dependent on their interaction with HSCs through the evolutionarily conservative ALK1-BMP9/10 axis.	(Guilliams et al. 2022
	ST-seq scRNA-seq	Tumor capsule prevents the infiltration of immune cells and thus redistributes immune cells around cancer. Moreover, tumor capsule affects intratumor spatial cluster continuity and transcriptome diversity.	(Wu et al., 2021
	scRNA-seq	Region-specific changes of LSECs in liver cirrhosis were indicated. Genes associated with capillarization of LSECs and extracellular matrix are mostly upregulated in LSECs of Zone 3. In addition, LSECs of Zone 3 also showed a decrease expression of endocytic receptor in cirrhotic mice. NO production-related transcription factors such as Klf2, Klf4 and AP-1 are down-regulated in LSECs of all zones in cirrhotic mice, indicating increased intrahepatic vascular resistance.	(Su et al. 2021

## Conclusions and perspectives

ScRNA-seq serves as a useful tool to decode the complexity of cell heterogeneity. On the basis of scRNA-seq, ST-seq further provides spatial information without the need of cell isolation. Moreso, ST-seq makes it possible to analyze RNA transcriptomes of specific cells in a specific region. However, the massive application of ST-seq is still limited by its low resolution. Fortunately, ST-seq with high resolution appears to be rapidly developing. A high-resolution spatial barcoding method-Seq-scope was developed, which can achieve submicrometer resolution (0.5 ~ 0.8 μm) (Cho et al. 2021). Compared to previous ST-seq, Seq-scope has dramatically increased its resolution by 3 orders of magnitude from 55 μm to 0.5 μm. In comparison with previous scRNA-seq data (Halpern et al. 2017), the authors confirmed the spatial distribution of key landmark genes. Furthermore, they can precisely locate NPCs distribution across the murine liver. Two populations of liver macrophages were identified. One is KCs highly expressing Clec4f and the other is inflammatory macrophages highly expressing CD74. They also found two populations of HSCs: one is normal HSCs (HSC-N) highly expressing actin alpha 2, smooth muscle (Acta2) and the other is activated HSCs (HSC-A) highly expressing extracellular matrix protein 1 (Ecm1). As well, they also found that injured hepatocytes (hep_injured) highly express serum amyloid A1 (Saa1). Both their sequencing analysis and histological staining indicated that with dead hepatocytes as the center, inflammatory macrophages and hep_injured cells are arranged around the periphery in turn. Generally, compared to previous ST-seq, Seq-scope can achieve much higher resolution and more precisely predict cell status in specific regions.

During the past few years, various technologies have been developed for spatial transcriptomics (Asp et al. 2020). These technologies include Geo-seq based on microdissection, multi-plex protein or mRNA staining, Slide-seq/Slide-seq V2, high-definition spatial transcriptomics (HDST), and Stereo-seq with high resolution and large areas. Geo-seq employs laser capture microdissection to capture cells of interest in situ, followed by library construction at the single-cell level. Since high-quality cDNA is produced to construct the library, high resolution could be achieved. However, throughput is relatively low. Slide-seq can reach single-cell resolution. This method uses a special chip preset with 10-μm beads with DNA barcodes. Its’ resolution is higher than ST-seq but unfortunately tissue imaging cannot be generated. Slide-seq V2 is similar to Slide-seq, but with an improved RNA capture efficiency. HDST is based on a randomly ordered bead array-based fabrication process. It deposits barcode sequences with ploy(d) T onto 2-μm magnetic beads onto glass slides and decodes the position information of each barcode through a sequential hybridization and error correcting strategy. The resolution is 1400 times higher than that of 10xVisium and 25 times higher than that of Slide-seq. Stereo-seq is a nano-level spatial transcriptome technology that combines a DNA nanoball chip and in situ capture technology, which has recently been applied to biological studies (Liu et al. 2022). With these advanced technologies applied to liver research, we believe our knowledge about the liver will be significantly increased in the near future.

Moreover, strengthening downstream analysis will also increase the use of ST-seq data (Dries et al. 2021). Therefore, further refinement and development of downstream analysis tools are needed. Simply put, downstream analysis tools can be divided into four categories. First, analysis tools to identify cell types. When ST-seq achieves single-cell resolution, unsupervised cluster tools such as Louvain and Leiden can be used to identify cell types. If ST-seq cannot reach single-cell resolution, cell type enrichment and spatial deconvolution tools such as RCTD, stereomicroscopy, Cell2location, Spatial DWLS, SPOTlight, DSTG, or DestVI can be used to identify cell types. Another complementary method for ST-seq analysis is to construct spatial information based on scRNA-seq data. Tomo-seq and Geo-seq can reconstruct 3D patterns from gene expression profiles obtained from 2D slices. Secondly, the improvement of analysis tools to characterize spatial patterns of transcriptomes are needed. Tools for this purpose include SpatialDE, SOMDE, Trendsceek, SPARK, and binSpect. Thirdly, analysis tools to analyze subcellular structure need to be improved. Tools that have been used for this purpose are SSAM, stLearn, Spage2vec, Sparcle, JTSA. Fourthly, improvement in analysis tools to understand cell communication under specific microenvironment needs to be improved. Cell behaviors can be significantly influenced by the tissue environment through direct physical interaction, secreted molecules, or interaction with extracellular matrix components. Giotto and CellPhoneDB are two resources that have been used to study cell communication. Moreover, Dynamo (Qiu et al. 2022) is a cutting-edge analytical framework, which can overcome the limitations of traditional splicing-based RNA velocity analysis. It can infer the absolute speed of RNA generation and degradation, reconstruct the continuous vector field, and finally predict cell trajectory. Tangram is a benchmarking spatial and single-cell transcriptomics integration method for transcript distribution prediction and cell type deconvolution. (Li et al. 2022) compared 16 methods that integrate scRNA-seq and ST-seq data and found that Tangram, gimVI, and SpaGE were better than other integration methods in predicting the spatial distribution of transcripts, while Cell2location, SpatialDWLS, and RCTD performed best in cell type deconvolution. Taken altogether, we believe development and improvement of analysis tool in the future will also promote the application of ST-seq. Cell state is regulated by many factors, such as mRNA, protein, post-translational modification and so on. Therefore, single cell proteomics or spatial proteomics might have a greater impact determining the identity and function of various liver cells. One of the difficulties in high throughput analysis of protein content via mass spectrometry is sample size. Lowly expressed proteins are highly likely to be missed. Moreover, post-translational modifications also play a significant role in regulation of cell function. These would be much harder to detect and are an area in urgent need for technical advancements. To fully understand the molecular mechanism in physiology or pathophysiology, all aspects of specific cellular alterations need to be considered. Researchers have been trying to obtain a more comprehensive multi-omic profile on tissues in order to deeply understand the complex regulation system (Guilliams et al. 2022; Liu et al. 2022). As mentioned above, single-cell CITE-seq, single-nuclei sequencing, spatial transcriptomics, and spatial proteomics have been integrated to generate a spatial proteogenomic atlas of the healthy and obese human and murine liver (Guilliams et al. 2022). The cell niche of lipid-associated macrophages (LAMs) was discriminated and localized. The data revealed the microenvironment circuits drive their unique transcriptome identity. As well, 91 zebrafish slices by stereo-seq were analyzed at six key time points during embryonic development (Liu et al. 2022). By integrating stereo-seq and scRNA-seq data, a developmental trajectory of cell fate transformation and molecular changes were reconstructed during this period. Moreover, potential important interactions were found by analyzing the spatial distribution of ligand/receptor pairs. The most feasible way to combine multiple “omes” is to process consecutive tissue sections and subject each tissue section to a different omic approach. Since spatial areas for each omic analysis will span multiple slices, data analysis will require a further integrative strategy. Ideally, if all omic analyses can be performed on the same tissue sample, data utilization and accuracy might be largely improved. Nonetheless, integrating transcriptomics, proteomics, and post translational modifications will be a big stride forward for liver research.

## Data Availability

Not applicable.

## Abbreviations

scRNA-seq: Single-cell RNA sequencing
ST-seq: Spatial transcriptome sequencing
NPCs: Non-parenchymal cells
LSECs: Sinusoidal endothelial cells
HSCs: Hepatic stellate cells
KCs: Kupffer cells
GO: Gene Ontology
KEGG: Kyoto Encyclopedia of Genes and Genomes
BECs: Bile duct epithelial cells
HHyP: Hepatobiliary hybrid progenitor
EpCAM: Epithelial cellular adhesion molecule
E: Embryonic day
PCW: Post-conception weeks
smFISH: Single molecule fluorescent in situ hybridization
Hamp: Hepcidin Antimicrobial Peptide
Igfbp2: Insulin Like Growth Factor Binding Protein 2
Mup3: Major urinary protein 3
Cyp8b1: Cytochrome P450 family 8 subfamily B member 1
CCA: Canonical correlation analysis
DGEA: Differential gene expression analysis
PaHSCs: Portal vein-associated HSCs
CaHSCs: Central vein-associated HSCs
CCl4: A carbon tetrachloride
Lpar1: Lysophosphatidic acid receptor 1
Mrc1: Mannose receptor C-type 1
Pecam1: Platelet and endothelial cell adhesion molecule 1
Kdr: Kinase insert domain receptor
Lyve1: Lymphatic vessel endothelial hyaluronan receptor 1
HPCs: Hepatocyte progenitor cells
APAP: Acetaminophen
TAA: Thioacetamide
ALF: Acute liver failure
AAs: ALF-activated stellate cells
AAe: ALF-activated endothelial cells
AAk: ALF-activated Kupffer cells
Sparcl1: Sparc like 1
Gja4: Gap junction protein alpha 4
LAMs: Lipid-associated macrophages
PLCs: Primary liver cancers
hcc: Hepatocellular carcinomas
chc-1: Cholangiocarcinoma
icc-1: Intrahepatic cholangiocarcinoma
MIA: Multimodal intersectional analysis
Klf2: Kruppel-like factor − 2
Klf4: Kruppel-like factor − 4
AP-1: Activation protein 1
Acta2: Actin alpha 2, smooth muscle
Ecm1: Extracellular matrix protein 1
Saa1: Serum amyloid A1
HDST: High-definition spatial transcriptomics
